# Self-measured leg circumference for the detection of lymphedema among men with prostate cancer: a reliability study

**DOI:** 10.2340/1651-226X.2025.42249

**Published:** 2025-02-27

**Authors:** Gitte Sone Larsen, Sandra Jensen, Annika von Heymann, Bolette Skjødt Rafn

**Affiliations:** Danish Cancer Society National Research Center for Cancer Survivorship and Treatment Late Effects (CASTLE), Department of Oncology, Copenhagen University Hospital Rigshospitalet, Copenhagen, Denmark

**Keywords:** Cancer-related lymphedema, prospective surveillance, home-based monitoring, leg lymphedema, psychometric properties

## Abstract

**Background and purpose:**

Early lymphedema detection is crucial to timely treatment, and home-based monitoring holds promise for early detection of leg lymphedema among at-risk cancer survivors. We developed a self-measurement protocol for home-based leg circumference measurements and tested its reliability in men with prostate cancer at risk of lymphedema.

**Patients/material and methods:**

This cross-sectional study recruited men with prostate cancer from the Department of Urology, Copenhagen University Hospital, Denmark. Circumference measurements were taken at four points on both legs, from which leg volume was calculated. Intrarater reliability was assessed by comparing self-measurements taken at home and in the hospital. Interrater reliability was evaluated by comparing hospital self-measurements to those of a blinded physiotherapist. Statistical power required 13 participants for the detection of a good (>0.8) intraclass correlation coefficient (ICC).

**Results:**

Forty-three men were included (median age 69 [63–76] years). Intrarater reliability (*n* = 39) was good to excellent for six out of eight measurement points (ICC ≥ 0.79, *p* < 0.01) and moderate for two (ICC ≥ 0.55, *p* < 0.01). Intrarater reliability for leg volume was excellent (ICC ≥ 0.96, *p* < 0.01). Similarly, interrater reliability (*n* = 23) was excellent for all measurement points and leg volumes (ICC ≥ 0.91, *p* < 0.01). Forty-one of 43 participants performed the measurements independently, found them easy to do, and were willing to conduct self-measurements if recommended by their doctor.

**Interpretation:**

Self-measured leg circumference among men with prostate cancer is highly reliable and acceptable. This low-cost approach for home-based monitoring for lymphedema offers potential for early detection and timely management of the condition.

## Introduction

Healthcare systems worldwide face increasing pressure from rising patient numbers and limited resources [1]. Consequently, there is a growing shift from hospital-based follow-up programs to home-based monitoring of health conditions, which has proven effective in managing conditions like diabetes by reducing hospital visits and improving patient outcomes [2]. The approach is now being explored for cancer-related late effects. In the case of lymphedema, which affects survivors of numerous cancer types [3], early detection is crucial to prevent long-term complications and reduce treatment costs [4], but requires repeated assessments using reliable outcome measurements.

Prospective surveillance with routine measurements of limb volume or extracellular fluid is recommended for cancer-related lymphedema [5]; however, assessment methods often require specialized equipment [6] and support from healthcare professionals, making them unsuitable for home settings. Home-based monitoring for lymphedema could offer significant benefits, such as earlier detection, improved cost-effectiveness, and reduced strain on patients and healthcare systems [7, 8].

While home-based monitoring of arm lymphedema using bioimpedance spectroscopy and circumference measurements has shown excellent reliability [9–11], leg lymphedema measurements, that is for patients with gynecological or prostate cancer, are typically conducted by healthcare professionals [12]. To date, one study has tested home-based leg circumference measurements. Brown et al. [13] reported moderate agreement (Cohen’s kappa 0.46) between home-based and nurse-reported measurements in women with endometrial cancer, with only 17 of 50 women completing the home-based measurements. This study used six measurement points, taken in a standing position, which 43% of participants found difficult to perform, thus limiting the utility of self-measurement.

For successful adoption of home-based lymphedema monitoring, simplicity, ease of use, and patient independence are essential. To meet these criteria, we developed a simple method for self-measurements of leg circumference using an inexpensive tape measure and intuitive instructions for home-based use.

The aim of this study was to assess the intra- and interrater reliability of self-measured leg circumference among men with prostate cancer. We hypothesized that the method would demonstrate high reliability, with intraclass correlation coefficients (ICC) of ≥0.8.

## Methods

### Participants

This study recruited men with prostate cancer through convenience sampling from the Department of Urology at Copenhagen University Hospital Rigshospitalet, Denmark, during January to March 2023. Adult patients in active treatment or follow-up who were Danish-speaking were eligible and invited by phone to participate.

### Development of measurement protocol

A protocol for self-measured leg circumference measurements was developed. The participant was in a sitting position with their knees and hips in 90-degree flexion. Measurements were taken at four points on both legs: 10 cm and 20 cm proximal to the patella (P10 and P20) and similarly 10 cm and 20 cm distal to the patella (D10 and D20) (Supplementary file 1). The measurement protocol was pilot tested on seven healthy volunteers and adjusted based on their feedback, including rephrasing text and replacing pictures for clarity.

### Procedures

Data collection occurred in three steps. In Step 1, hospital-based measurements were performed. Participants first performed self-measured leg circumference following the protocol, with verbal guidance from a physiotherapist if needed. In Step 2, an experienced physiotherapist who was blinded to the self-measurements performed therapist-measurements at the same four points. Step 3 involved home-based self-measurements conducted one or two days after Step 1. Participants were provided with a printed measurement protocol, a tape measure, and a measuring stick to facilitate home-based self-measurements. The time of measurement was not standardized, rather measurements were conducted at times during the day that was convenient for participants. Participants either reported their measurements in an electronic questionnaire or via telephone to the research staff.

### Outcome measures

Leg circumference measurements were reported in centimeters (cm). The truncated cone formula was used to determine the volume in milliliters (mL) of the proximal (P10–P20) and the distal (D10–D20) part of the leg. These two were added to get a proxy of the total leg volume.


$$V=\frac{h(C_{1}^{2}+C_{1}\cdot C_{2}+C_{2}^{2}}{12\pi }$$

where *V* = volume, *h* = length of the cone, C_1_ = proximal circumference, and C_2_ = distal circumference.

Standard error of measurement (SEM) and minimal detectable change (MDC) were calculated for all circumference measurements and the total leg volume. The SEM was calculated using the standard deviation (SD) of the difference of means between the first and second self-measurements: SEM = SD_difference_/√2. The 95% MDC was calculated using the formula MDC = 1.96*SEM*√2. For the total leg volume, SEM and MDC were expressed as percentages: SEM/mean*100 and MDC/mean*100.

In Step 1, participants reported demographic variables (i.e. body mass index (BMI), civil status, income, employment, and educational level) and answered three questions: (1) Did you conduct the measurements yourself? (yes/no); (2) Would you be willing to measure your legs at home if your doctor told you that you were at risk of developing lymphedema? (yes/no); and (3) How easy or difficult was it to measure your legs? (Scale ranging from 1 “very difficult” to 10 “very easy”). The clinical variables (i.e. age, time since diagnosis, cancer stage, and treatment type) were collected from medical records.

### Statistical analysis

Intrarater and interrater reliability, which assess the consistency and agreement of measurements within and between raters, were analyzed using the ICC two-way mixed effect, absolute agreement, and single measurement (ICC 2,1).


$$ICC(2,1)=\frac{MS_{R}-MS_{E}}{MS_{R}+(k-1)MS_{E}+\frac{k}{n}(MS_{C}\_MS_{E})})$$

where MS_R_ = mean square for rows, MS_E_ = mean square for error, MS_C_ = mean square for columns, *n* = number of subjects, and *k* = number of rater/measurements.

ICC values with 95% confidence intervals (CIs) and p-values were reported for all measurement points (cm) and total leg volumes (mL) for both legs. ICC values range from 0 to 1, with <0.5 indicating poor reliability, 0.5–0.75 moderate, 0.75–0.9 good, and >0.9 excellent reliability [14]. All analyses were performed in R (version 4.3.0).

Outliers were included in the main analysis to reflect real-world variability [15], and additional sensitivity analyses excluding outliers using the Tukey’s fences [16] were performed.

### Sample size

Based on the hypothesis of detecting ICC of ≥ 0.8 for intrarater and interrater reliability, 13 participants were required (α = 0.05, 80% power) [15].

### Ethics

The Danish Data Protection Agency (R-22071980) and The Capital Region of Copenhagen Ethical Committee judged that ethical approval was not required (F-23004790). The Helsinki Declaration was followed for all aspects of the study. Participants signed an informed consent form before entering the study.

## Results

A total of 43 men participated, with a median age of 69 [interquartile range (IQR) 63–76] years and a median BMI of 26 [IQR 23–29] kg/m^2^. Participants had stages I–IV prostate cancer with a median time since diagnosis of 5 [IQR 1–8] years. They had received different combinations of treatments and represented a range of education, income, and employment levels (Table 1).

**Table 1:** Characteristics of participants.

Characteristics	*n* = 43
Age, *years*, median [interquartile range (IQR)]	69 [63–76]
BMI, *kg/m^2^*, median [IQR]	26 [23–29]
Civil status, *n* (%)	
*Cohabiting partner*	31 (72)
* Living alone*	12 (28)
Highest education, *n* (%)	
*Primary school*	7 (16)
*Vocational education*	14 (33)
*Short education 2–3 years*	3 (7)
*Undergraduate education 3–4 years*	10 (23)
*Graduate education > 4 years*	9 (21)
Employment status, *n* (%)	
*Full time >30 h/week*	7 (16)
*Part time ≤ 30 h/week*	5 (12)
*Retired*	24 (56)
*Sick leave*	1 (2)
*Other*	6 (14)
Family income, *n* (%)	
*100.000–250.000 dkk*	9 (21)
*250.000–400.000 dkk*	13 (30)
*400.000–550.000 dkk*	4 (9)
*550.000–700.000 dkk*	4 (9)
*700.000–850.000 dkk*	3 (7)
*850.000–1.000.000 dkk*	4 (9)
*>1.000.000 dkk*	6 (14)
Time since diagnosis, median [IQR]	5 [1–8]
Cancer stage, *n* (%)	
*I*	6 (14)
*II*	18 (42)
*III*	9 (21)
*IV*	10 (23)
Treatment type, *n* (%) *More than one treatment type is possible	
* Prostastectomy*	22 (51)
*Prostatectomy with lymph node removal*	14 (33)
*Hormone therapy*	23 (54)
*Chemotherapy*	9 (21)
*Radiation*	10 (23)
*Active surveillance or watchful waiting*	8 (19)

BMI: body mass index.

The results demonstrated good to excellent intrarater reliability (*n* = 39) for the self-measurements for six out of eight measurement points (ICCs ≥ 0.79). The measurement points 20 cm distal to the patella (D20) on both legs showed moderate intrarater reliability (ICCs ≥ 0.55). The intrarater reliability of the volumes of the legs was excellent (ICC ≥ 0.96). There was excellent interrater reliability (*n* = 23) for all measurement points and for total leg volume between self-measurements and therapist-measurements (ICCs ≥ 0.91) (Table 2). The MDC ranged from 2.1 to 7.4 cm on the right leg, and the MDC for the volume of the leg was 237 mL, corresponding to 9% (Table 3). Similarly, for the left leg, the MDC ranged from 2.7 cm to 6.7 cm, and the MDC for the leg volume was 274 mL, corresponding to 10%. The sensitivity analyses showed excellent reliability for all points and improved ability to detect change (Supplementary File 2).

**Table 2:** Intrarater reliability of self-measured leg circumference (*n* = 39) and interrater reliability between self-measurements and therapist-measurements (*n* = 23).

Point of measure	Type of reliability	Right				Left			

		ICC	95% CI	*p*	Reliability	ICC	95% CI	*p*	Reliability
P20 (cm)	Intrarater	0.83	0.70–0.91	<0.01	Good	0.83	0.71–0.91	<0.01	Good
	Interrater	0.96	0.90–0.98	<0.01	Excellent	0.98	0.95–0.99	<0.01	Excellent
P10 (cm)	Intrarater	0.95	0.90–0.97	<0.01	Excellent	0.95	0.91–0.97	<0.01	Excellent
	Interrater	0.98	0.95–0.99	< 0.01	Excellent	0.99	0.98–1.00	<0.01	Excellent
D10 (cm)	Intrarater	0.94	0.89–0.97	<0.01	Excellent	0.79	0.64–0.88	<0.01	Good
	Interrater	0.98	0.94–0.99	<0.01	Excellent	0.99	0.97–0.99	<0.01	Excellent
D20 (cm)	Intrarater	0.55	0.30–0.74	<0.01	Moderate	0.66	0.44–0.81	<0.01	Moderate
	Interrater	0.91	0.75–0.96	<0.01	Excellent	0.96	0.91–0.98	<0.01	Excellent
Volume (mL)	Intrarater	0.97	0.95–0.99	<0.01	Excellent	0.96	0.93–0.98	<0.01	Excellent
	Interrater	0.99	0.99–1.00	< 0.01	Excellent	0.99	0.99–1.00	<0.01	Excellent

Measurement points: P20: 20 cm proximal to the base of patella; P10: 10 cm proximal to the base of patella; D10: 10 cm distal to the apex of patella; D20: 20 cm distal to the apex of patella. Volume: Total leg volume.

**Table 3:** Minimal detectable change for self-measured leg circumference (*n* = 39).

Measure point	Right leg			Left leg		
	SD_difference_	SEM	MDC	SD_difference_	SEM	MDC
P20 (cm)	3.3	2.4	6.6	3.2	2.3	6.4
P10 (cm)	1.3	0.9	2.6	1.4	1.0	2.7
D10 (cm)	1.1	0.8	2.1	2.3	1.6	4.4
D20 (cm)	3.8	2.7	7.4	3.4	2.4	6.7
Volume (mL)	120.8	85.4	236.7	139.8	98.8	274.0
Volume (%)		3.1	8.7		3.7	10.1

SD_difference_: Standard deviation of the difference of the means between hospital and home measurements; SEM: Standard error of the mean; MDC: Minimal detectable change for home self-measured circumference of the lower limbs. Measurement points: P20: 20 cm proximal to the base of patella; P10: 10 cm proximal to the base of patella; D10: 10 cm distal to the apex of patella; D20: 20 cm distal to the apex of patella. Volume: Total leg volume.

Forty-one of 43 participants (95%) reported that they conducted the home-based self-measurements independently, and that they would conduct home-based self-measurements if recommended by their doctor (1 response was missing). The participants rated the ease of performing home-based self-measurements to a median of 9.5 [IQR 8–10].

## Discussion

The results of our study demonstrated excellent intra- and interrater reliability of self-measured leg circumference and leg volumes among men with prostate cancer. Participants’ measurements were reliable when tested against those of an experienced physiotherapist and when performed on two separate occasions. Participants conducted the measurements independently, found the protocol easy to follow, and were willing to conduct home-based self-measurements if recommended by their doctors. This high acceptance highlights the potential for empowering patients to take an active role in their health management, which can alleviate the burden on patients and healthcare facilities.

The implementation of reliable home-based monitoring for lymphedema addresses inequities in healthcare access. Patients living in rural areas or with limited mobility face barriers to accessing healthcare facilities for lymphedema monitoring [17]. Early detection of lymphedema is crucial, as it enables timely intervention and better clinical outcomes [8]. Remote monitoring provides a viable alternative, enabling these patients to manage their health effectively without frequent hospital visits. This is important, given the growing population of cancer survivors and the current lack of systematic lymphedema surveillance in most countries. In addition to economic benefits, home-based monitoring fosters greater patient engagement and adherence to treatment regimens, further supporting its implementation in clinical settings [2, 18]. The excellent reliability of self-measurements demonstrated in this study is a promising first step toward home-based monitoring of leg lymphedema. However, further research is needed to investigate the sensitivity and specificity of this approach and its ability to ensure early detection.

To our knowledge, only Brown et al. [14] have investigated self-measured leg circumference, testing a protocol among women with endometrial cancer. No study has explored this approach among men with cancer. While Brown et al. did not assess the ability to detect changes in leg volume, this is a critical aspect of effective lymphedema detection. The MDC represents the smallest detectable change that reflects a true change, excluding measurement error. In our study, we found MDCs of 9 and 10% for leg volumes. While direct comparisons are difficult due to differing methodologies, Jönsson et al. reported lower MDCs (<4%) among men and women with and without leg lymphedema [12, 19], based on leg circumference measurements by healthcare professionals. Clinical thresholds for lymphedema diagnosis range from 5 to 20% volume changes [20]. While some studies recommend thresholds of 5–10% for minimal lymphedema [21, 22], a recent study suggested raising the threshold to 15% (compared to pre-surgery measurements) due to the fluctuating nature of the condition [23]. Using the commonly applied 10% threshold [24–26], our measurement method could detect clinically relevant changes in leg volume, potentially signaling the onset of lymphedema. While difference in time of measurement in our study could theoretically lead to daytime bias as leg volume may increase during the day [27], our results suggest that self-measurements are reliable even when performed at a time that is convenient to each person.

The ICC values at D20 showed moderate reliability, suggesting higher variability, potentially due to changes in limb position or tape measure reading errors. Refining the self-measurement protocol, such as incorporating a video guide demonstrating the procedure, as used by Rafn et al. [9, 11] and suggested by participants in Brown et al. [13], could potentially enhance accuracy. These refinements could support reliable self-measurements without requiring in-person teaching.

A key strength of this study is the development of a written and illustrated protocol for self-measurement of leg circumference that was easy to understand and perform using inexpensive tools, thus holding potential for home-based monitoring for lymphedema. Another strength is that our sample included men living alone (28%) and men with diverse levels of income, education, and employment, as well as different cancer stages and treatment types, thus suggesting that this approach is feasible to perform for people with varying resources. Future studies should use a prospective design with repeated self-measurements, ideally starting with baseline measurements before prostate cancer treatment to assess feasibility in urology departments and the method’s ability to detect leg volume changes over time. Additionally, validation against other diagnostic methods, such as perometry, should also be explored.

## Conclusion

Self-measured leg circumference among men with prostate cancer is highly reliable and acceptable. Furthermore, this approach allows for the detection of small changes in leg volume, which could be indicative of lymphedema. More work is needed to investigate the potential of this low-cost approach for home-based monitoring in enabling early detection of leg lymphedema among this large group of survivors.

## Supplementary Material

Self-measured leg circumference for the detection of lymphedema among men with prostate cancer: a reliability study

## Data Availability

Data are available upon reasonable request to the corresponding author.
