# Supplementary material for: Self-measured leg circumference for the detection of lymphedema among men with prostate cancer: a reliability study
Source: Acta Oncol. 2025 Feb 27;64:42249. doi: 10.2340/1651-226X.2025.42249 (PMC11884415; doi:10.2340/1651-226X.2025.42249)
Supplement: Self-measured leg circumference for the detection of lymphedema among men with prostate cancer: a reliability study [file AO-64-42249-s1.pdf]

Kræftens Bekæmpelses Nationale Forskningscenter for Senfølger hos Kræftoverlevere, medlem af DCCC
CASTLE

Supplementary material has been published as submitted. It has not been copyedited, or typeset by Acta Oncologica

Supplementary file 1: Self-measurement protocol

Sit on a chair with bare legs. You need to mark the measurement points on both legs

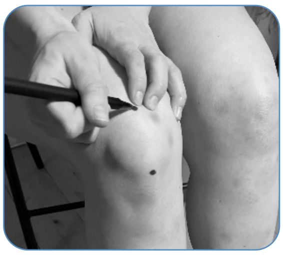

Mark the upper and lower edge of the kneecap with a dot

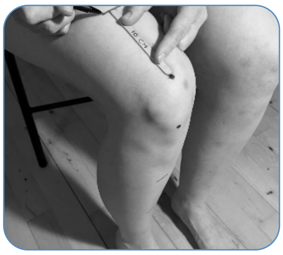

Place the 10 cm measurement stick at the dot above the kneecap and draw a line as a measurement point.

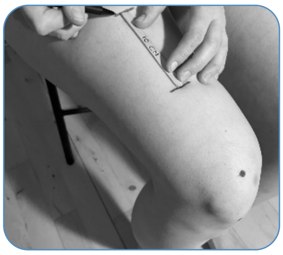

Place the measurement stick at the new point and mark another measurement point with a line

- You now have two measurement points on your thigh
- One 10 cm above and one 20 cm above the kneecap

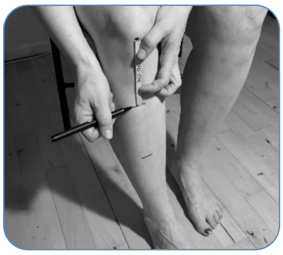

Place the measurement stick at the dot below the kneecap and draw a line as a measurement point on your shin.

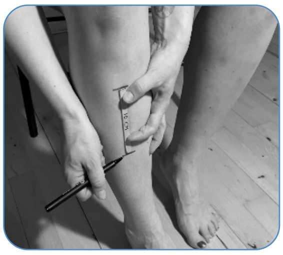

Place the measurement stick at the new point and mark another measuremnt point with a line.

- You now have two measurement points on you shin
- One 10 cm below and one 20 cm below the kneecap

Kræftens Bekæmpelses Nationale
Forskningscenter for Senfølger hos
Kræftoverlevere, medlem af DCCC
CASTLE

You will now measure four points on each leg. Measure the two upper and the two lower points. Do not measure the dots at the kneecap.

Sit with your knee bent at a 90° during the measurement.

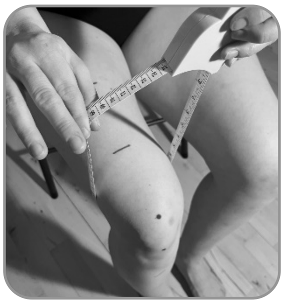

Pool the tape measure and place it around your leg.

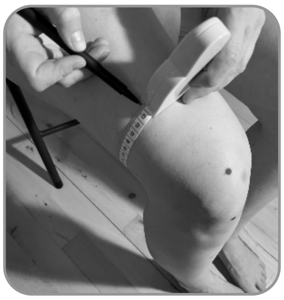

Place the white "box" under the desired measurement point. Press the button to tighten the tape measure

- Read the measurement directly, or mark the tape for easier reading
- Record the measurement on the figure below

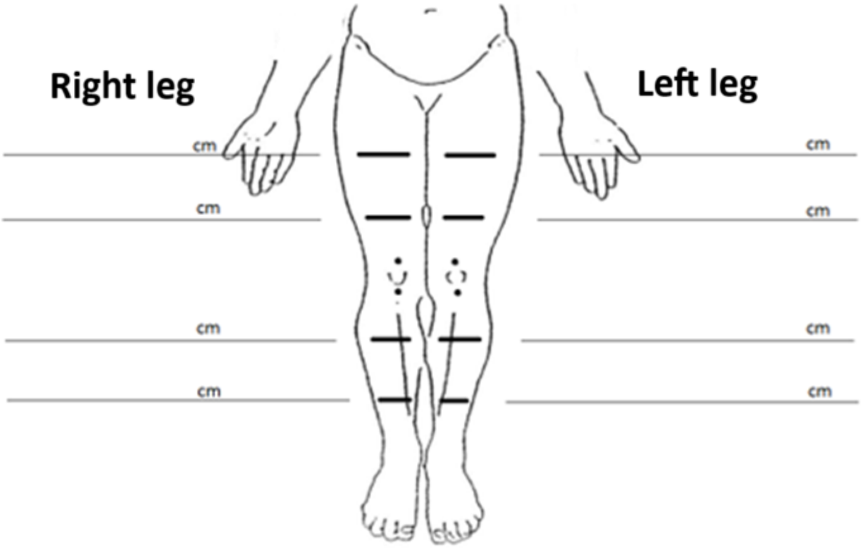

How easy or difficult was it to measure your legs? (Rate on a scale from 1 to 10)

Very difficult 1 2 3 4 5 6 7 8 9 10 Very easy

Did you conduct the measurements yourself? Yes / no

Would you be willing to measure your legs at home if your doctor told you that you were at risk of developing lymphedema? Yes / No

Supplementary file 2: Sensitivity analyses

Sensitivity analyses of intra- and interrater reliability (Table A), as well as standard error of measurement and minimal detectable change (Table B), was conducted to evaluate the impact of excluding outliers.

Table A: Sensitivity analysis of intrarater reliability of self-measured leg circumference and interrater reliability between self-measurements and therapist measurements, conducted excluding outliers.

Measurement point	Reliability	Right leg		Left leg	
		ICC	95%CI	ICC	95%CI
P20 (cm)	Intrarater	0.95	0.91 - 0.98	0.95	0.90 - 0.97
	Interrater	0.97	0.89 - 0.99	0.98	0.96 - 0.99
P10 (cm)	Intrarater	0.95	0.90 - 0.97	0.95	0.91 - 0.97
	Interrater	0.98	0.96 - 0.99	0.99	0.98 - 1.00
D10 (cm)	Intrarater	0.96	0.91 - 0.98	0.92	0.84 - 0.96
	Interrater	0.98	0.94 - 0.99	0.99	0.97 - 1.00
D20 (cm)	Intrarater	0.87	0.76 - 0.93	0.93	0.87 - 0.97
	Interrater	0.97	0.85 - 0.99	0.97	0.93 - 0.99
Volume (mL)	Intrarater	0.98	0.95 - 0.99	0.96	0.93 - 1.00
	Interrater	0.99	0.97 - 1.00	0.99	0.99 - 1.00

Measurement points: P20 = 20cm promixal to the base of patella, P10 = 10cm proximal to the base of patella, D10 = 10 cm distal to the apex of patella, D20 = 20 cm distal to the apex of patella. Volume = Total leg volume
ICC: Intraclass correlation coefficient

Table B: Sensitivity analysis of minimal detectable change, conducted excluding outliers.

Measurement point	Right leg			Left leg		
	$SD_{difference}$	SEM	MDC	SDdifference	SEM	MDC
P20 (cm)	1.9 cm	1.0 cm	2.9 cm	1.5 cm	1.1 cm	3.0 cm
P10 (cm)	1.3 cm	0.9 cm	2.6 cm	1.4 cm	1.0 cm	2.7 cm
D10 (cm)	0.9 cm	0.6 cm	1.7 cm	1.2 cm	0.9 cm	2.4 cm
D20 (cm)	2.0 cm	1.4 cm	3.8 cm	1.3 cm	0.9 cm	2.5 cm
Volume (mL)	107.8 mL	76.2 mL	211.2 mL	139.8 mL	98.8 mL	274.0 mL
Volume (%)		2.7%	7.8%		3.7%	10.1%

$SD_{difference}$ = Standard deviation of the difference of the means between hospital and home measurements. SEM = Standard error of the mean. MDC = Minimal detectable change for home self-measured circumference of the lower limbs. Measurement points: P20 = 20cm proximal to the base of patella, P10 = 10cm proximal to the base of patella, D10 = 10 cm distal to the apex of patella, D20 = 20 cm distal to the apex of patella. Volume = Total leg volume.
